# COVID-19 outbreak in Brazil: adherence to national preventive measures and impact on people’s lives, an online survey

**DOI:** 10.1186/s12889-021-10222-z

**Published:** 2021-01-18

**Authors:** Edlaine Faria de Moura Villela, Rossana Verónica Mendoza López, Ana Paula Sayuri Sato, Fábio Morato de Oliveira, Eliseu Alves Waldman, Rafael Van den Bergh, Joseph Nelson Siewe Fodjo, Robert Colebunders

**Affiliations:** 1 School of Medicine, Health Sciences Unit, Federal University of Jataí, Jataí, Brazil; 2 Center for Translational Research in Oncology, Institute of Cancer of São Paulo State, São Paulo, Brazil; 3 Department of Epidemiology, School of Public Health, University of São Paulo, São Paulo, Brazil; 4 Global Health Institute, University of Antwerp, Antwerp, Belgium

**Keywords:** Prevention, Behavior, pandemics, Public health, surveillance, COVID-19, South America

## Abstract

**Background:**

The first case of COVID-19 infection was diagnosed in Brazil 26th February 2020. By March 16th, physical distancing and confinement measures were implemented by the Brazilian government. Little is known about how these measures were followed up by the Brazilian people and their impact on daily routine.

**Methods:**

In early April 2020, using an online platform, we organized an online survey among adults living in Brazil about their COVID-19 preventive behavior and impact on their daily routine.

**Results:**

Data from 23,896 respondents were analyzed (mean age: 47.4 years). Due to COVID-19 restrictions, half (51.1%) of the professionals reported working from home. Regular handwashing was practiced by 98.7% of participants; 92.6% reported adhering to the 1.5-2 m physical distancing rule, but only 45.5% wore a face mask when going outside. While 29.3% of respondents found it relatively easy to stay at home, indoor confinement was extremely difficult for 7.9% of participants. Moreover, 11% of participants were extremely worried about their health during the COVID-19 epidemic. Younger people, male, persons living in a rural area/village or popular neighbourhoods, students and workers reported less preventive behaviour.

**Conclusion:**

Restrictive measures markedly affected the daily and professional routines of Brazilians. Participants showed a satisfactory level of adherence to national COVID-19 prevention guidelines. Qualitative and follow-up studies are needed to monitor the impact of COVID-19 in the Brazilian society.

**Supplementary Information:**

The online version contains supplementary material available at 10.1186/s12889-021-10222-z.

## Background

On December 31st 2019, the World Health Organization (WHO) received a notification of an unknown viral pneumonia outbreak in the Hubei Province of China. This outbreak was later found to be caused by the Severe Acute Respiratory Syndrome Coronavirus 2 [1, 2]. The disease, now called Coronavirus Disease 2019 (COVID-19), has quickly spread to most countries of the world, affecting almost 5 million individuals and causing more than 320,000 deaths. Until May 22, 492,124 cases were registered in the South America, with 55,3% being in Brazil [3].

COVID-19 is primarily transmitted by respiratory droplets with a similar incubation time and development time as the previously known Severe Acute Respiratory Syndrome Coronavirus (SARS-CoV) [1, 4]. The rapid international spread of COVID-19 pressured the WHO to declare the COVID-19 epidemic as a public health emergency of international importance in late January 2020. Such a decision is taken when an event with major public health implications crosses the borders of the country initially affected, demanding immediate international action [5]. In the absence of antivirals and while awaiting the large-scale introduction of COVID-19 vaccination [6], various public health strategies to contain the infection have been implemented around the world. These strategies commonly consist of enforced or semi-enforced “lockdowns” and closure of national and/or intra-national borders, as well as promotion of respiratory hygiene (masking, coughing/sneezing etiquette) and hand hygiene. The package of containment measures for COVID-19 around the world probably represents the largest global public health intervention in human history, though the societal and individual impact of these measures is not yet well-understood.

The population-level adherence to such measures may determine to a considerable extent the national magnitude and duration of the COVID-19 pandemic [7, 8]. However, little is known on population-level adherence to the various containment measures implemented worldwide, with most studies focusing on adherence to hygiene measures among healthcare workers [9–11]. In-depth documentation of adherence to containment measures is nonetheless essential, on the one hand to feed into initiatives attempting to model outbreaks [7, 12], and on the other hand to adapt and target health promotion messages to sub-populations that may be struggling to adhere to specific measures [13], such as specific age groups.

In Brazil, the first case of COVID-19, reported by the Ministry of Health (MOH) on February 26th, was a 61-year-old man who had traveled to Italy between February 9 and 21 of 2020. Two tests were positive for COVID-19 infection. Since then, the number of infected persons in Brazil has increased dramatically [14]. Physical distancing and confinement measures were implemented by the Brazilian government after COVID-19 was declared a pandemic on March 16th [5]. Events expected to attract large numbers of people were cancelled, universities and schools were closed, and only services considered essential to the population remained functional, such as markets, pharmacies and bakeries. However, traveling between Brazilian states remained possible. To document how the containment measures affected the lives of the Brazilian people, and to understand which containment measures were best adhered to by which strata of the population, we conducted an online survey on the adherence of the Brazilian people to individual public health measures and impact of the COVID-19 outbreak on people’s lives. A particular emphasis was placed on age as a stratifying factor, considering the clear association of COVID-19 severity with age, and the general need for adapting health messaging to specific age groups.

## Materials and methods

### Study design

An online questionnaire survey was organized in Brazil between April 3 to April 9. At the time of the survey Brazil counted 9056 confirmed COVID-19 cases, 1769 hospitalizations and 359 deaths. Data were collected on the ICPcovid website (www.icpcovid.com), which is a secure web-based platform developed by the University of Antwerp, Belgium.

The link for the survey was disseminated using WhatsApp, email, and social media such as Facebook, Instagram, LinkedIn and ResearchGate. Furthermore, we had support from national organizations such as the Research Support Foundation of the State of Goiás, the Regional Council of Biology of the First Region, and the Faculty of Public Health of the University of São Paulo who actively disseminated the survey link. Everybody in Brazil, regardless their nationality could participate in the study: the only exclusion criteria for participation were being younger than 18 years (as we considered that approval from one of the parents was needed) and not living in Brazil at the moment of the survey.

The structured questionnaire consisted of 60 questions about socio-demographic characteristics, individual preventive measures (such as hand and respiratory hygiene, physical distancing and isolation) and daily living practices (such as impact on working conditions, difficulties to adhere to the preventive measures and COVID-19 health concerns); See supplemental information. We used Likert scores for questions concerning health risk perception and the level of difficulty to observe the preventive measures.

### Data analysis

Statistical analysis was performed using IBM SPSS version 25 for Windows. Containment measures were grouped into three main categories: hand hygiene, respiratory hygiene, and physical distancing/isolation. A composite adherence score was generated for each of these categories using specific questions from the survey, with empirical weights (Table 1). Subsequently an overall adherence score was generated by combining the sub-scores using equal weights (1:1:1 ratio).

**Table 1:** Composite adherence score to COVID-19 preventive measures

***Preventive measures***	Composite adherence score
***Hand hygiene***	
Wash hands regularly with water and soap OR with alcohol gel	1
Avoid touching face	0.5
Disinfect cell phone	0.5
	*Divide total score by 2*
***Respiratory hygiene***	
Wear a face mask when leaving home	1
Covering face or nose with forearm or tissue when sneezing or coughing	1
Wash hands after coughing/sneezing	1
	*Divide total score by 3*
***Physical distancing/isolation***	
Follow rule of staying 1.5-2 m from other people	1
Measure temperature twice a week	0.5
Stay home when experiencing flu-like symptoms (among people who had flu-like symptom days)	1
	*Divide total score by 2.5*
**Overall composite score across all measures (1:1:1 ratio of specific scores)**	

Descriptive statistics were presented using means with standard deviation (SD) for continuous outcomes, and percentages (%) for categorical variables. The Wilcoxon test was used to compare the number of days of work per week before and after the epidemic.

Multiple linear regression was performed to analyze factors associated with adherence to national prevention restrictive measures; the composite adherence score (Table 1) served as dependent variable. Variables with *p* < 0.10 in bivariate analysis were included in the adjusted model, and the final model was selected via a backward stepwise process of eligible covariates. Covariates that were investigated included: age, gender, state and area of residence, education, marital status, living alone, profession, working in the health sector, and existing comorbidities. The significance level adopted was 5% for all hypothesis tests.

## Results

### Characteristics of respondents

A total of 25,266 persons participated in the survey. After excluding respondents younger than 18 years (*n* = 163) and people with inconsistent responses (1207), 23,896 respondents (94.6%) were included for analysis. Participants were from all parts of the country. The median age of participants was 48.0 years (IQR 37.0–58.0 years); 71.8% were women (Table 2). 7020 (29.4%) reported a chronic underlying disease such as diabetes, cancer, HIV infection or tuberculosis and 2177 were smokers of cigarettes.

**Table 2:** Characteristics of study participants in an online survey on COVID-19, Brazil, April 2020

***Characteristics***	***N*** = 23,896		
***Continuous variables***			
*Age in years*			
	Median (Q1-Q3)	48 (37–58)	
	Range	18–89	
***Categorical variables***		**N**	**%**
*Age group*	*18–25 years*	1652	6.9
	*26–65 years*	20,109	84.2
	*> 65 years*	2135	8.9
*Gender*	Male	6741	28.2
	Female	17,155	71.8
* Brazilian region of residence*	North	299	1.3
	Northeast	2315	9.7
	Central-West	2489	10.4
	Southeast	13,447	56.3
	South	3428	14.3
	Not answered / missing data	1918	8.0
* Nationality*	Brazilian	23,746	99.4
	Foreign	150	0.6
*Highest educational level*	I didn’t complete elementary school	1	0.0
	Primary School	99	0.4
	Secondary School	2437	10.2
	University Undergraduate degree holder	7604	31.8
	University Postgraduate degree holder	13,755	57.6
*Marital status*	Single	5876	24.6
	Legally married	12,167	50.9
	Cohabitation	2556	10.7
	Divorced	2713	11.4
	Widow/widower	584	2.4
*Residential setting*	Downtown area	13,046	54.6
	Suburb area	4531	19.0
	Rural area/village	631	2.6
	Popular neighborhoods	5688	23.8

### Impact of COVID-19 restrictive measures on working arrangements

At the time of the survey, 44.6% of professionals were working from home. For those who were not working from home, 66.1% were not able to do so because of the type of job, 9.1% were not allowed by their employer, 5% had to leave the house to make money to support the family, and 1.3% left the home because they considered this to be without a risk (Table 3). Due to COVID-19 restrictions, participants reported going to work less often (mean number of days of work per week: 0.8) compared to the period before the epidemic (mean number of days of work per week: 3.2; *p*-value< 0.001; Wilcoxon test).

**Table 3:** Impact of COVID-19 restrictions on working conditions

Characteristics	Description	n	%
Profession (*n* = 23,896)	Unemployed	938	3.9
	Student	1551	6.5
	Self-employed	5235	21.9
	Work for the government (federal, state, municipal)	7028	29.4
	Work for a person, institution or company	5200	21.8
	Other	3944	16.5
*Healthcare worker* (n = 23,896)	Yes	7293	30.5
	No	16,603	69.5
*Current working conditions (n = 21,407 workers)*	Work from home	9544	44.6
	Work in an open space (market, shop, roadside, etc)	1452	6.8
	Work in a closed indoor space with several people (office, etc.)	5614	26.2
	Work in a closed indoor space alone (office, etc.)	1833	8.6
	Not informed	2964	13.8
*Reasons for not working from home (n = 5931 workers)*	It is not possible with my job	3918	66.1
	It is possible, but is not allowed by my employer	540	9.1
	I don’t think there is any risk to go out	76	1.3
	I have to leave the house to make money to support my family	298	5.0
	Other	1099	18.5

### Adherence to the national COVID-19 restrictions

Most participants (92.6%) reported adhering to the 1.5-2 m social distancing rule; 69.5% covered their mouth and nose when they sneeze and washed their hands afterwards; 45.5% wore a face mask when going outside; Staying at home was found to be extremely difficult for 7.9%, but 29.3% considered it not difficult at all (Table 4).

**Table 4:** Adherence to national anti-COVID-19 preventive measures

	n = 23,896	N	%
*I follow the social 1.5-2 m meters distance rule*	Yes	22,117	92.6
*I wear a face mask when going outside*	Yes	10,876	45.5
Covering face or nose with forearm or tissue when sneezing or coughing	Yes	22,515	94.2
*When I cough/sneeze, I usually wash/disinfect my hands soon after*	Yes	16,618	69.5
*I measure my body temperature at least twice a week*	Yes	2586	10.8
*I wash my hands using soap and water regularly during the day*	Yes	23,591	98.7
*I use a hand sanitizer regularly during the day*	Yes	17,758	74.3
*I avoid touching my face (eyes, nose and mouth)*	Yes	18,549	77.6
*I disinfect my phone whenever I return home*	Yes	16,454	68.9
*I travelled to another city/country during the last 7 days*	Yes	1339	5.6
*Individual difficulty level to adhere to the national preventive measures for COVID-19 (1 = not difficult at all to 5 = extremely difficult)*			
	1	6990	29.3
	2	5651	23.6
	3	6249	26.2
	4	3113	13.0
	5	1893	7.9

### Difficulty to adhere to the COVID-19 preventive measures and health concerns

**Fig. 1:**
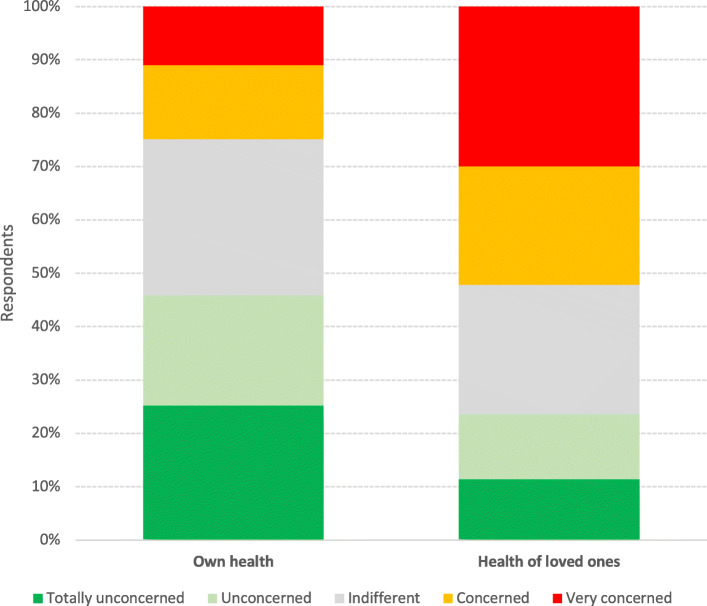
Level of concern about COVID-19 consequences among participants of an online survey on COVID-19, Brazil, April 2020 (bivariate: self-versus loved ones)

While 29.3% of respondents found it relatively easy to stay at home, indoor confinement was extremely difficult for 7.9% of participants (Table 3). When queried about their health concerns as a consequence of COVID-19, respondents were more concerned about the health of their loved ones (29.9% very concerned and 22.2% concerned) than their own health (11.0% very concerned and 13.9% concerned) (Fig. 1).

Multiple linear regression analysis was carried out to identify factors associated with higher overall adherence. Older age, being female, having at least an undergraduate degree, being a health care worker, having comorbidities, not living in the rural area/village, not being a student, not working in the private sector, and not smoking were all independently associated with a higher overall score (Table 5).

**Table 5:** Factors associated with adherence to national prevention restrictive measures

Variable	Categories	B	Standard Error	95% CI	***p***-value
*n* = 23,896					
	Intercept	1.743	0.0473	1.650–1.836	< 0.001
Age	*(in years)*	0.007	0.0002	0.006–0.007	< 0.001
Gender	Male				
	Female	0.128	0.0063	0.116–0.141	< 0.001
Education	Primary School or less				
	Secondary School	0.059	0.0445	−0.028-0.146	0.184
	Undergraduate	0.099	0.0439	0.013–0.186	0.023
	Postgraduate	0.141	0.0439	0.055–0.227	0.001
Area of residence	Downtown area				
	Suburban area	−0.007	0.0075	−0.022-0.006	0.295
	Rural area/village	−0.086	0.0178	− 0.117--0.048	< 0.001
	Popular neighborhoods	−0.004	0.0071	−0.018-0.009	0.552
Profession	Unemployed				
	Student	−0.058	0.0186	−0.095--0.022	0.002
	Self-employed	0.011	0.0156	−0.018-0.042	0.453
	Work for the government (federal, state, municipal)	−0.022	0.0154	−0.052-0.008	0.150
	Work for a person, institution or company	−0.045	0.0155	−0.075--0.015	0.003
	None of the previous	0.002	0.0164	−0.029-0.034	0.885
Health care worker	No				
	Yes	0.044	0.0065	0.031–0.056	< 0.001
Smoking	No				
	Yes	−0.024	0.0098	−0.043--0.0047	0.015
Comorbidities	Not that I know				
	Yes	0.030	0.0064	0.017–0.043	< 0.001

**Fig. 2:**
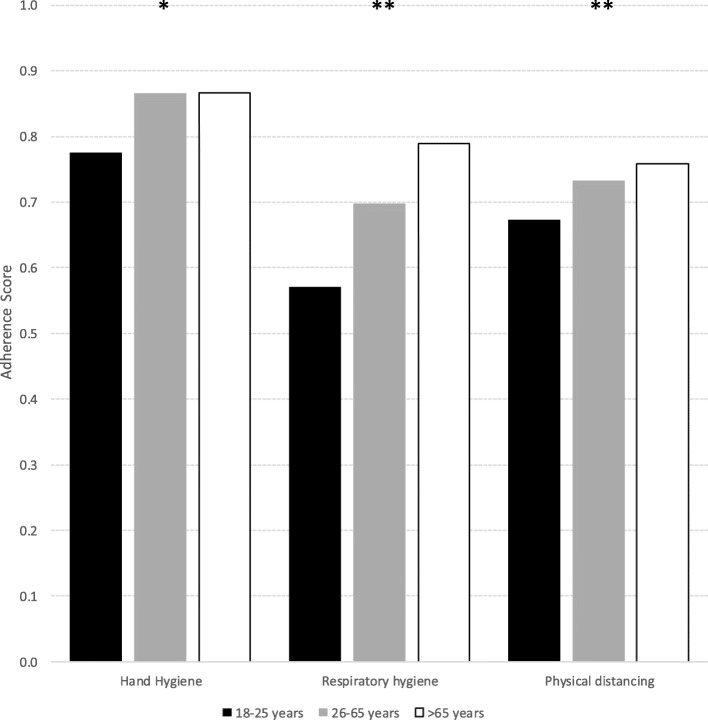
Scores for adherence to COVID-19 containment measures, among respondents of an online survey on COVID-19, per age group, Brazil, April 2020. * *p* < 0.001 for 18–25 years vs 26–65 years and > 65 years. ** *p* < 0.001 for 18–25 years vs 26–65 years and > 65 years; 26–65 years vs > 65 years

Adherence scores for the specific measures were all significantly lower in the younger age group (18–25 years). Respiratory hygiene and physical distancing adherence scores were significantly lower in the 26–65 years old than in the > 65 years old – confounding of the other covariates was controlled for through multiple linear regression (Fig. 2).

## Discussion

Our study shows that, during the survey period, Brazilians were following the COVID-19 preventive measures relatively well. Hand hygiene measures were adhered to most, followed by physical distancing and respiratory hygiene. In all categories of measures, a clear age effect was observed, with younger individuals scoring lower than older respondents on the adherence score.

Overall, only 45.5% reported wearing a face mask when going out. This is much lower than in Asian countries, where most people wore face masks once the COVID-19 epidemic was introduced in their country [15–17]. This is also lower than the 91.7% face mask use reported in a similar online survey in Ecuador in April 2020 and the 99.8% face mask use in a survey in Peru in June 2020 [18]. This is however higher than in several European countries where wearing face masks was initially advised only in health care settings, following WHO recommendations at the time [19–21]. Checking one’s temperature for the early detection of a COVID-19 infection at least twice a week was only practiced by 10.8% of the respondents. This may be a point of concern, as WHO reported that temperature screening was able to detect the majority of exported cases during the COVID-19’s expansion [22].

When assessing the profile of individuals with poor general adherence, men were less adherent compared to women, which mirrors findings from a Knowledge, Attitudes, and Practices study conducted in China (17). In our study, younger age was also associated with a lower overall score. In another large online survey in Brazil conducted between April 24th and May 24th, assessing only the degree of adherence to physical contact restriction measures, greater adherence was found among females compared to males but it was mainly the 30 to 49 year old group that was less adherent and not the younger age group [23]. People living in rural areas and poor neighborhoods were also less adherent: in rural areas people may not perceive themselves at high risk of COVID-19, and therefore may not respect the national restriction measures and not practice individual hygiene measures [24]. Therefore, extra communication and health education may be needed to change the risk perception in rural areas and popular neighborhoods [25]. Brazilian students reported difficulties to stay home, which may be related to a need to travel to their original homes in periods when schools and universities were closed [26] or could be related to differing social habits among this population. Encouragingly, respondents with underlying diseases followed the preventive measures well, which is important considering their higher risk for more severe disease.

Taken together, these observations suggest that tailoring of the public health messages may be indicated. A reinforcement of specific messages, such as mask use and temperature taking, may be beneficial, and using delivery methods tailored to the specific age groups could allow higher uptake. Especially communication methods to the younger age group could benefit from such tailoring, and possibly approaches relying on social media and including influencers to spread public health messages could be considered [27]. Of note, the observation that most respondents’ concern was higher for their loved ones than for themselves could be incorporated in such health messages; possibly by emphasizing how adhering to measures protects one’s close environment.

In general, our results indicate that following an intensive COVID-19 prevention campaign [28] the Brazilians gradually became aware of the importance of adopting simple methods to prevent COVID-19 transmission. For only 7.9% of the respondents indoor confinement was experienced as extremely difficult. Initially the MOH of Brazil expected a peak of COVID-19 infections during the second half of April. However, it did not happen. The satisfactory adherence to the preventive measures may have delayed the peak of the epidemic.

COVID-19 associated mortality during the study period was highest in the North region of Brazil (Amazonas) and in two states in the Northeast (Ceará and Pernambuco) [29]. Our study showed that the Northeast region had less difficulty to adhere the restrictive measures. This difference between regions may have been influenced by the adoption of restrictive measures to varying degrees by the governors of the Brazilian states. Indeed, 11 states have decreed lockdown for at least one municipality in their state. Only the state of Amapá decreed a lockdown for all your municipalities.

There has been a lot of confusion about how to deal with the COVID-19 epidemic in Brazil. The president has minimized the actions of the MOH, downplaying the importance of quarantine, and is defending vertical isolation to avoid financial collapse. Vertical isolation or shielding implies that most people return back to normal life and people with underlying diseases, older adults and pregnant women continue to respect physical distance and reduce their social activities. Regarding this vulnerable group, 29.4% stated to have underlying diseases in our survey. This is a concern, as older age and the presence of (an) underlying health condition(s) are associated with increased COVID-19 related mortality [30, 31] On the other hand, Brazilian respondents with underlying diseases adhered better to the containment measures.

The lack of unified actions against COVID-19, by the federal government, led to the resignation of the health minister on April 16 [32, 33]. From that moment on, there was a relaxation of quarantine measures, opening of part of the trade, and consequently less physical distancing. The lack of national coordination by the government in response to the pandemic reveal the conflictual positions between the federal government and governors from the 27 states of the country [34]. This increased the number of COVID-19 cases and associated deaths [30, 31].

At the end of April, the COVID-19 death toll in Brazil had already exceeded that of China [3] (more than 5000 deaths) and this scenario got worse, not reaching the flattening of the curve and overloading the Brazilian health system [35]. As of December 31th 2020, more than 7,000,000 cases had been confirmed in the country, causing almost 200,000 deaths [3].

Our findings suggest a considerable initial willingness of the Brazilian people to follow the quarantine and other containment measures. However, this willingness seems to have been irrevocably subverted through the political stance against the public health measures with as a consequence that currently South America became the new epicenter of the pandemic with Brazil as the most affected country [3].

Our study had several limitations**.** The number of respondents was relatively small compared to the entire Brazilian population, and respondents were unevenly spread over the national territory. Indeed, only 2,6% of the participants reported residing in rural areas. The reason for this low number of participants from rural areas most likely is because in those areas people have less internet access and consequently are less linked to social media [24, 25]. While 51.8% of the Brazilian population are women [36], 71,8% of the respondents in our survey were female. Such a higher proportion of female respondents was also observed in other studies on COVID-19-related practices [17]. Participants were more likely to be higher educated individuals living in cities and in the Southeast region. The latter may be explained by the fact that since the beginning of the pandemic, this region recorded the largest number of COVID-19 infections. Moreover, broadband internet quality is best in the Southeast region [37]. Our survey was also not able to reach vulnerable populations, such as the homeless, prisoners, older adults, migrants and people with mobility problems. Such populations may be at increased risk for COVID-19 infection and should be considered as priority key groups in the prevention and control of Covid-19 [26, 38]. Our study findings are based on self-reports without a possibility to verify whether these responses corresponded with the real preventive behaviour of the respondents.

At the time of writing, the COVID-19 vaccination started in more than 30 countries [39], and in Brazil, it is scheduled to start at the end of January 2021. However, it will still be challenging to deal with the vaccine hesitance movements and the political polarization [40] that it is taking place in relation to vaccination.

## Conclusion

In conclusion, most participants in this survey correctly followed the COVID-19 prevention guidelines, although staying at home was difficult for individuals who had to go out because of their job, and younger individuals tended to adhere less to containment measures. Larger follow-up surveys and in-depth qualitative studies about the preventive behavior of different groups in the Brazilian society are needed. The adherence to COVID-19 preventive measures will need to be monitored closely as restrictive measures are being relaxed and as the expectations concerning the COVID-19 vaccine may decrease the motivation of people to adhere to prevention measures.

## Supplementary Information

**Additional file 1.** Survey questionnaire

## Data Availability

The datasets used and/or analysed during the current study are available from the corresponding author on reasonable request.

## Abbreviations

COVID-19: Coronavirus Disease 2019
MOH: Ministry of Health
SD: Standard deviation
WHO: World Health Organization

## References

[1] Zhu N, Zhang D, Wang W (2020). A novel coronavirus from patients with pneumonia in China, 2019. N Engl J Med.

[2] Gorbalenya AE, et al. Severe acute respiratory syndrome-related coronavirus: the species and its viruses – a statement of the coronavirus study group. Microbiology. 2020.

[3] World Health Organization. WHO Coronavirus Disease (COVID-19) Dashboard Available at: <https://covid19.who.int>. Access on: 31 dec 2020.

[4] Wilson ME, Chen LH. Travelers give wings to novel coronavirus (2019-nCoV). J Travel Med. 2020. 10.1093/jtm/taaa015 PMC7107561 32010938

[5] World Health Organization. Coronavirus disease 2019 (COVID-19). Situational Report-37. Disponível em: <https://www.who.int/docs/default-source/coronaviruse/ situation reports/20200226-sitrep-37-covid-19.pdf>. Accessed 27 Feb 2020.

[6] Regmi K, Lwin CM (2020). Impact of non-pharmaceutical interventions for reducing transmission of COVID-19: a systematic review and meta-analysis protocol. BMJ Open.

[7] Acuña-Zegarra MA, Santana-Cibrian M, Velasco-Hernandez JX. Modeling behavioral change and COVID-19 containment in Mexico: A trade-off between lockdown and compliance [published online ahead of print, 2020 May 6]. Math Biosci. 2020:108370. 10.1016/j.mbs.2020.108370. 10.1016/j.mbs.2020.108370 PMC7202859 32387384

[8] Jarvis CI, Van Zandvoort K, Gimma A (2020). Quantifying the impact of physical distance measures on the transmission of COVID-19 in the UK. BMC Med.

[9] Houghton C, Meskell P, Delaney H (2020). Barriers and facilitators to healthcare workers' adherence with infection prevention and control (IPC) guidelines for respiratory infectious diseases: a rapid qualitative evidence synthesis. Cochrane Database Syst Rev.

[10] Hillier MD (2020). Using effective hand hygiene practice to prevent and control infection. Nurs Stand.

[11] Saitoh A, Sato K, Magara Y (2020). Improving Hand Hygiene Adherence in Healthcare Workers Before Patient Contact: A Multimodal Intervention in Four Tertiary Care Hospitals in Japan. J Hosp Med.

[12] Ngonghala CN, Iboi E, Eikenberry S (2020). Mathematical assessment of the impact of non-pharmaceutical interventions on curtailing the 2019 novel coronavirus [published online ahead of print, 2020 may 1]. Math Biosci.

[13] West R, Michie S, Rubin GJ, Amlôt R (2020). Applying principles of behaviour change to reduce SARS-CoV-2 transmission. Nat Hum Behav.

[14] BRASIL. Ministério da Saúde. Secretaria de Vigilância em Saúde. Centro de Operações de Emergências em Saúde Pública. Boletim Epidemiológico Especial 14. 2020. Available online: www.saude.gov.br/bvs. Acessed on 30 Apr 2020.

[15] Wang Q, Yu C (2020). The role of masks and respirator protection against SARS-CoV-2. Infect Control Hosp Epidemiol.

[16] Noh JY, Seong H, Yoon JG, Song JY, Cheong HJ, Kim WJ (2020). Social Distancing against COVID-19: Implication for the Control of Influenza. J Korean Med Sci.

[17] Zhong BL, Luo W, Li HM (2020). Knowledge, attitudes, and practices towards COVID-19 among Chinese residents during the rapid rise period of the COVID-19 outbreak: a quick online cross-sectional survey. Int J Biol Sci.

[18] Siewe Fodjo JN, Pengpid S, Villela EFM, Van Thang V, Ahmed M, Ditekemena J, Crespo BV, Wanyenze RK, Dula J, Watanabe T, Delgado-Ratto C, Driessche KV, Van den Bergh R, Colebunders R (2020). Mass masking as a way to contain COVID-19 and exit lockdown in low- and middle-income countries. J Inf Secur.

[19] Hernández-García I, Giménez-Júlvez T (2020). Assessment of Health Information About COVID-19 Prevention on the Internet: Infodemiological Study. JMIR Public Health Surveill.

[20] Geldsetzer P (2020). Use of Rapid Online Surveys to Assess People's Perceptions During Infectious Disease Outbreaks: A Cross-sectional Survey on COVID-19. J Med Internet Res.

[21] Feng S, Shen C, Xia N, Song W, Fan M, Cowling BJ (2020). Rational use of face masks in the COVID-19 pandemic. Lancet Respir Med.

[22] World Health Organization. Available online: https://www.who.int/news-room/articles-detail/updated-who-advice-for-international-traffic-in-relation-to-the-outbreak-of-the-novel-coronavirus-2019-ncov-24-jan . Accessed on 24 May 2020.

[23] Szwarcwald CL, Souza Júnior PRB, Malta DC, Barros MBA, Magalhães MAFM, Xavier DR (2020). Adesão às medidas de restrição de contato físico e disseminação da COVID-19 no Brasil. Epidemiol Serv Saúde.

[24] Prusaczyk B. Strategies for Disseminating and Implementing COVID-19 Public Health Prevention Practices in Rural Areas [published online ahead of print, 2020 Apr 3]. J Rural Health. 2020. 10.1111/jrh.12432. 10.1111/jrh.12432 32246497

[25] Ranscombe P (2020). Rural areas at risk during COVID-19 pandemic. Lancet Infect Dis.

[26] Wang P, Lu J-A, Jin Y, Zhu M, Wang L, Chen S. Statistical and network analysis of 1212 COVID-19 patients in Henan, China. Int J Infect Dis. 2020. 10.1016/j.ijid.2020.04.051 PMC7180361 32339715

[27] Kuno Crative. Available online: https://www.kunocreative.com/blog/influencers-doing-good-during-covid-19. Accessed on 24 May 2020.

[28] BRASIL. Ministério da Saúde. Secretaria de Vigilância em Saúde. Centro de Operações de Emergências em Saúde Pública. Plano de Contingência Nacional para Infecção Humana pelo novo Coronavírus COVID-19. 1. ed. 2020. Available online: www.saude.gov.br/bvs. Accessed on 24 Apr 2020.

[29] Mellan TA, Hoeltgebaum HH, Mishra S (2020). Estimating COVID-19 cases and reproduction number in Brazil. Imperial College London.

[30] Giacomelli A (2020). 30-day mortality in patients hospitalized with COVID-19 during the first wave of the Italian epidemic: a prospective cohort study. Pharmacol Res.

[31] Asfahan S, et al. Extrapolation of mortality in COVID-19: Exploring the role of age, sex, co-morbidities and health-care related occupation. Monaldi Arch Chest Dis. 2020;90(2). 10.4081/monaldi.2020.1325 PMID: 32447949. 10.4081/monaldi.2020.1325 32447949

[32] Valente, J. Covid-19: uso maior da internet requer mais cuidado com segurança. Agência Brasil. 2020. Available online: https://agenciabrasil.ebc.com.br/saude/noticia/2020-03/covid-19-uso-maior-da-internet-requer-mais-cuidado-com-seguranca. Accessed on 30 Apr 2020.

[33] COVID-19 in Brazil: "So what?". [editorial]. The Lancet. 2020; 395(10235):1461. 10.1016/S0140-6736(20)31095-3 PMC7251993 32386576

[34] Matos CCSA, Barbieri CLA, Couto MT (2020). Covid-19 and its impact on immunization programs: reflections from Brazil. Rev Saude Publica.

[35] Requia WJ, et al. Risk of the Brazilian health care system over 5572 municipalities to ex- ceed health care capacity due to the 2019 novel coronavirus (COVID-19). Sci Total Environ. 730(2020):139144. 10.1016/j.scitotenv.2020.139144 PMC7252142 32380368

[36] IBGEeduca. Portal do IBGE voltado para educa√ß√£o. Conhe√ßa o Brasil: Popula√ß√£o - Quantidade de homens e mulheres. Available online: https://educa.ibge.gov.br/jovens/conheca-o-brasil/populacao/18320-quantidade-de-homens-e-mulheres.html. Accessed on 03 Jan 2021.

[37] Comitê Gestor da Internet no Brasil. Núcleo de Informação e Coordenação do Ponto BR. Banda larga no Brasil: um estudo sobre a evolução do acesso e da qualidade das conexões à Internet /[coordenação executiva e editorial Alexandre F. Barbosa]. São Paulo: Available online: https://cetic.br/media/docs/publicacoes/1/Estudo%20Banda%20Larga%20no%20Brasil.pdf. Accessed on 24 Apr 2020.

[38] Lima CKT (2020). The emotional impact of coronavirus 2019-nCoV (new coronavirus disease). Psychiatry Res.

[39] El País. Pandemia de coronavirus. Mais de 30 países iniciaram vacinação contra covid-19, e Bolsonaro agora fala em pressa por vacina. Available online: https://brasil.elpais.com/brasil/2020-12-27/mais-de-30-paises-iniciaram-vacinacao-contra-covid-19-e-bolsonaro-agora-fala-em-pressa-por-vacina.html. Accessed on 03 Jan 2021.

[40] Guimarães R (2020). Vacinas Anticovid: um Olhar da Saúde Coletiva. Ciênc. saúde coletiva [Internet].

