# Supplementary material for: COVID-19 outbreak in Brazil: adherence to national preventive measures and impact on people’s lives, an online survey
Source: BMC Public Health. 2021 Jan 18;21:152. doi: 10.1186/s12889-021-10222-z (PMC7812554; doi:10.1186/s12889-021-10222-z)
Supplement: Supplementary file 1 — Additional file 1. Survey questionnaire [file 12889_2021_10222_MOESM1_ESM.docx]

**How do you deal with COVID-19?**

*- ICPCovid questionnaire -*

**Introduction**

The new coronavirus (SARS-CoV-2) is a virus that infects individuals around the world and can cause respiratory problems and in some cases, death. The disease caused by the virus was named COVID-19. The SARS-CoV-2 is already circulating in Brazil, and a lot of efforts are being invested to prevent its spread in our community. In that light, the Brazilian government took a number of measures to prevent the spread of coronavirus. With this short questionnaire, we try to find out how you experience and apply the instructions implemented against coronavirus. We call upon volunteers to complete this questionnaire as truthfully as possible; this helps us investigate which measures are effective in these exceptional times. It should take you about 10 minutes to answer the questions. Participation in the survey is voluntary, you can cancel it at any time without any disadvantages. Your data will be stored anonymously and treated confidentially.

This is an online, voluntary survey initiated by an international group of researchers from Asian, African, South American and European countries. We do not require your personal information, and the data will be used to understand the feasibility and effectiveness of implementing preventive measures for the coronavirus at individual level. The survey is anonymous and hosted on a secure server. If you would like to know more about the researchers involved in this study and our privacy policy, you can find this on the www.ICPcovid.com website.

**PART 1: Socio-demographic information**

1. Age

2. Sex

Male

Female

3. Nacionality

I am a Brazilian

I am a foreigner

4. In which Brazilian region do you live?

North

Northeast

Midwest

Southeast

South

5. In which Brazilian state do you live?

6. Religion

Christian (Catholic. Protestant. Adventist. Spiritist. Jehovah's Witness. among others)

Islamic

Jewish

Buddhist

Another religion

None

7. What is your maximum educational level?

I didn't complete elementary school

Primary School

Secondary School

University Undergraduate degree holder

University Postgraduate degree holder

8. Marital status

Single

Legally married

Cohabitation

Divorced

Widow/widower

9. With whom you live? (Many responses are possible)

With my parents

With my spouse/partner

With my child(ren)

My siblings (s) or other relative

With friends

Alone

10. How many housemates do you have (yourself not included)? Please write the number of people within each age group.

Adults over 70 years of age

Adults between 18 and 70 years of age

Children between 12 and 17 years of age

Children under 12 years of age

11. Where do you live?

Downtown area

Suburb area

Rural area/village

Popular neighborhoods

12. What are your housing conditions?

Apartment without balcony

Simple house (without slab. without floor) or hut

Single storey house or townhouse with backyard

Single storey house or townhouse without backyard

A room or kitnet

Homeless

**PART 2: Daily life during the coronavirus epidemic**

1. How many people apart from your housemates did you talk to yesterday face to face (not by phone, chat etc)?

2. When was the last time you shook hands, gave a kiss or had any form of physical contact with someone other than a housemate?

Today

Last two days

Last 3 to 5 days

More than one week ago

3. During the last week did you have difficulties in obtaining food?

Yes

No

4. During the last week, how worried or afraid were you about your health?

1 = not worried to 5 = extremely worried

1

2

3

4

5

5. Have you suffered any form of violence or discrimination because of the measures taken against the coronavirus?

Physical violence at home

Physical violence outside

Discrimination because of my social/economic status

Discrimination because of my ethnicity, race or nationality

No violence or discrimination

6. How did you arrange the care of your children today?

At friends / acquaintances

At aunts or uncles

At grandparents

At home, with my housemates

At home, with a house help/nanny

At home, by myself

To school / childcare

Other

Not Applicable (no children at home)

**PART 3: Professional life during the coronavirus epidemic**

1. What do you do for a living?

Unemployed

Student

Self-employed

Work for the government (federal, state, municipal)

Work for a person, institution or company

None of the previous

2. Are you a healthcare worker or a student working in the health sector?

Yes

No

3. What are your current working conditions?

Work from home

Work in an open space (market, shop, roadside, etc)

Work in a closed indoor space with several people (office, ….. etc.)

Work in a closed indoor space alone (office, ….. etc.)

Does not apply (if unemployed or student)

4. How many days per week do you usually go to school or work?

5. How many days did you (physically) go to school or work last week?

6. Are you working from home today?

Yes

No

Does not apply (if unemployed or student)

7. If no, why are you not working from home?

It is not possible with my job

It is possible, but is not allowed by my employer

I am at home but not working

I don’t think there is any risk to go out

I have to leave the house to make money to support my family

Other

Does not apply

8. What transportation means did you use to go to work?

Hired a vehicle for myself and/or family members (private taxi, rented car, etc)

Own transport (vehicle, motorcycle, bicycle)

By public transport with multiple people (train, bus, taxi, Uber, etc)

Walked to work

Does not apply

**PART 4: Individual preventive measures for COVID-19**

1. Do you follow the social 1.5-2m meters distance rule?

Yes

No

2. Do you wear a face mask when going outside?

Yes

No

3. When you cough or sneeze, do you cover your mouth and nose with a tissue paper or into your elbow?

Yes

No

4. When you cough or sneeze, do you usually wash/desinfect your hands immediately afterwards?

Yes

No

5. Do you measure your body temperature at least twice a week?

Yes

No

6. Do you wash your hands using soap and water regularly during the day?

Yes

No

7. Do you use a hand sanitizer regularly during the day?

Yes

No

8. Do you avoid touching your face (eyes, nose and mouth)?

Yes

No

9. Do you disinfect your phone whenever you return home?

Yes

No

10. Do you stay home when you feel flu-like symptoms?

Yes

No

11. How difficult is it for you personally to follow the protective measure of staying home as much as possible? (1 = not difficult at all <=> 5 = extremely difficult)

1

2

3

4

5

**PART 5: Community preventive measures for COVID-19**

1. Approximately how many times did you wash your hands or use a hand sanitizer yesterday?

2. Were you in a meeting or gathering with more than 10 persons during the last 7 days?

Yes

No

3. Did you go to a restaurant, bar, club, dancing, party, or concert during the last 7 days?

Yes

No

4. Did you go to a religious gathering during the last 7 days?

Yes

No

5. Did you go to a funeral during the last 7 days?

Yes

No

6. Were you in a vehicle or bus with more than 5 persons during the last 7 days?

Yes

No

7. Were you in a gym during the last 7 days?

Yes

No

8. Did you go to a beauty parlour. massages. spa. hairdresser or nail studio during the last 7 days?

Yes

No

9. Did you go to a market during the last 7 days?

Yes

No

10. Have you been using common plates or spoons when eating together with family members during the last 7 days?

Yes

No

11. Have you been using common plates or spoons when eating together with non-family members during the last 7 days?

Yes

No

12. Did you travel in the past 7 days?

No travel

Yes, I traveled to another municipality in the same state

Yes, I traveled to another state

Yes, I traveled outside the country

13. During the last week. how worried or afraid were you about the health of your loved ones? (1= totalmente despreocupado; 2= despreocupado; 3= indiferente; 4= preocupado; 5=muito preocupado)

1

2

3

4

5

14. On a scale of 1 to 10. can you indicate the extent to which people in your environment have practically adapted their behavior to the government recommendations? (1 = no adaptations. 10 = very strong adaptation)

0

1

2

3

4

5

6

7

8

9

10

**PART 6: Questions related to your personal health**

1. Have you been eating more healthy food such as fruits and vegetables since the coronavirus epidemic started?

Yes

No

2. Have you been taking more vitamin tablets since the coronavirus epidemic started?

Yes

No

3. Did you have flu-like symptoms in the last 7 days (cough or sore throat, shortness of breath, headaches, body pains, fever, loss of taste or smell)?

Yes

No

Do not know

4. For how many days did you have flu-like symptoms?

5. Are these symptoms still present?

Yes

No

6. Have any of your housemates had flu-like symptoms in the last 7 days?

Yes

No

Do not know

7. Do you smoke?

Yes

No

8. Do you have an underlying disease (e.g. heart disease, asthma, diabetes, hypertension, cancer, HIV, tuberculosis, etc)?

Yes

Not to my knowledge

9. If you have an underlying disease did you experience difficulties to obtain your medication since the coronavirus outbreak started?

Yes

No

I fully understand what this study is about, and I consent to participate. All the information I provide can be used by researchers to better understand coronavirus disease in my country.

**Thank you very much for your participation.**

We would like to invite you to participate in another survey in a few weeks. Participation is voluntary and optional. For further information about the Covid-19 situation in Brazil, please visit the website of the Ministry of Public Health at: www.saude.gov.br
